# Correction to “Spatiotemporal Trend and Hotspot Analysis of Stillbirth Prevalence in Ghana”

**DOI:** 10.1002/hsr2.73186

**Published:** 2026-09-04

C. Boateng, M. A. Ofori, S. Mintah, et al., “Spatiotemporal Trend and Hotspot Analysis of Stillbirth Prevalence in Ghana,” *Health Science Reports* 9, no. 7 (2026): e72742, https://doi.org/10.1002/hsr2.72742.

In the References section, reference [27] was incorrect and should be replaced with the following reference:

27. J. A. Akuu, M. A. Amagnya, “Community‐Based Management of Acute Malnutrition: Implementation Quality, and Staff and User Satisfaction With Services,” *Journal of Taibah University Medical Sciences* 18, no. 5 (2023): 988–996, https://doi.org/10.1016/j.jtumed.2023.02.002.

We apologize for this error.

